# Tryptophan (W) at position 37 of murine IL-12/IL-23 p40 is mandatory for binding to IL-12Rβ1 and subsequent signal transduction

**DOI:** 10.1016/j.jbc.2021.101295

**Published:** 2021-10-09

**Authors:** Jacqueline Georgy, Yvonne Arlt, Jens M. Moll, Meryem Ouzin, Hendrik T. Weitz, Lothar Gremer, Dieter Willbold, Joachim Grötzinger, Felix Thives-Kurenbach, Jürgen Scheller, Doreen M. Floss

**Affiliations:** 1 Institute of Biochemistry and Molecular Biology II, Medical Faculty, Heinrich-Heine-University Düsseldorf, Düsseldorf, Germany; 2 Institute of Biological Information Processing (IBI-7: Structural Biochemistry) and JuStruct: Jülich Center for Structural Biology, Forschungszentrum Jülich, Jülich, Germany; 3 Institut für Physikalische Biologie, Heinrich-Heine-Universität Düsseldorf, Düsseldorf, Germany; 4 Institute of Biochemistry, Medical Faculty, Christian-Albrechts-University Kiel, Kiel, Germany

**Keywords:** IL-12, IL-23, IL-12Rβ1, IL-23R, IL-12Rβ2, STAT, ERK, signal transduction, protein–protein interaction, site 1-2-3 paradigm, HIL-6, Hyper-IL-6, HIL-12, Hyper-IL-12, HIL-23, Hyper-IL-23, IL, interleukin, IL-12Rβ1, interleukin 12 receptor β1, IL-12Rβ2, interleukin 12 receptor β2, IL-23R, interleukin 23 receptor

## Abstract

Interleukin (IL)-12 and IL-23 are composite cytokines consisting of p35/p40 and p19/p40, respectively, which signal *via* the common IL-12 receptor β1 (IL-12Rβ1) and the cytokine-specific receptors IL-12Rβ2 and IL-23R. Previous data showed that the p40 component interacts with IL-12Rβ1, whereas p19 and p35 subunits solely bind to IL-23R and IL-12Rβ2, resulting in tetrameric signaling complexes. In the absence of p19 and p35, p40 forms homodimers and may induce signaling *via* IL-12Rβ1 homodimers. The critical amino acids of p19 and p35 required for binding to IL-23R and IL-12Rβ2 are known, and two regions of p40 critical for binding to IL-12Rβ1 have recently been identified. In order to characterize the involvement of the N-terminal region of p40 in binding to IL-12Rβ1, we generated deletion variants of the p40-p19 fusion cytokine. We found that an N-terminal deletion variant missing amino acids M23 to P39 failed to induce IL-23-dependent signaling and did not bind to IL-12Rβ1, whereas binding to IL-23R was maintained. Amino acid replacements showed that p40W37K largely abolished IL-23-induced signal transduction and binding to IL-12Rβ1, but not binding to IL-23R. Combining p40W37K with D36K and T38K mutations eliminated the biological activity of IL-23. Finally, homodimeric p40D36K/W37K/T38K did not interact with IL-12Rβ1, indicating binding of homodimeric p40 to IL-12Rβ1 is comparable to the interaction of IL-23/IL-12 and IL-12Rβ1. In summary, we have defined D36, W37, and T38 as hotspot amino acids for the interaction of IL-12/IL-23 p40 with IL-12Rβ1. Structural insights into cytokine–cytokine receptor binding are important to develop novel therapeutic strategies.

The interleukin (IL-)12 type cytokines IL-12 and IL-23 play critical roles in a variety of innate and acquired immune responses. Misregulation eventually causes chronic autoimmune responses, and Ustekinumab, an antibody blocking IL-12/IL-23 signaling, was recently approved for psoriasis, psoriatic arthritis, Crohn’s disease, and ulcerative colitis (1). Knowledge about structure and composition of the IL-12/IL-23 cytokine:receptor complexes, as well as signaling components, offered new possibilities for effective targeting strategies of both cytokines. In general, cytokines of the IL-6/IL-12 family activate the JAK/STAT pathway, the MAPK pathway, and the PI3K/AKT pathway. Albeit, STAT1, 3, 4, and 5 are activated by IL-12 and IL-23, STAT4 remains the dominant signaling molecule for IL-12 and STAT3 for IL-23 (2).

IL-12 cytokines function as heterodimers comprised of α and β subunits. IL-12 and IL-23 share their β subunit p40, which is linked to α subunits through disulfide bonds formed between C199 of p40 and C96 of p35 or C73 of p19 for human IL-12 and IL-23, respectively (3, 4, 5). Interactions of IL-12 type cytokines with their cognate receptors follow the “site 1-2-3” structural model based on the structure of IL-6, IL-6R and gp130 (6, 7, 8). p40 uses the same binding site 1 of domains 2 and 3 to interact with both p19 and p35 (9). Aside from the central cysteine residues, R207 and Y211 in murine p35, R211 and Y215 in human p35 are critical for association with p40 (10). For murine IL-23, mutations in I176, A178, and R179 of p19 prevent the formation of IL-23 (11).

The receptor complex interfaces between the cytokine subunits of IL-12/IL-23, and the respective receptors IL-12Rβ1 and IL-12Rβ2 or IL-23R are named site 2 and site 3, respectively. Based on the structure of IL-23, our work showed that murine p19W157 is the hotspot amino acid for site 3 interaction with murine IL-23R (9, 11). For p40, the domains D2 and D3 are also required to correctly position the binding site 3 of IL-23 to IL-23R (11). In 2018, the crystal structure of human IL-23:IL-23R in complex with a single-domain VHH camelid antibody targeting human IL-23 (Nb22E11) was solved (12). This finding allowed us to show that combined substitutions of W156A and L160E result in an inactive human p19 cytokine variant on cells expressing human IL-23 receptors. For site 3 interaction between IL-12 and IL-12Rβ2, we demonstrated that the p19-analogous site 3 substitution Y189R in human p35 abolishes binding to IL-12Rβ2 in a cross-species manner (13).

Typically, site 2 is mediated by the cytokine α subunit. However, interaction of p19/p35 with IL-12Rβ1 is unlikely. Several data suggest that p19/p35 are dispensable for this interaction and that rather p40 directly interacts with IL-12Rβ1. First of all, secreted p40 binds directly to IL-12Rβ1 and is an effective antagonist of IL-12 and IL-23 (14, 15, 16). Additionally, p40 monomers require IL-12Rβ1, but not IL-12Rβ2, to attenuate autoimmune diseases (17). Second, IL-12 or IL-23 signaling is blocked by Ustekinumab. Ustekinumab binds to an epitope on domain 1 of human p40 consisting of three loops (W37-M45, L62-L69, T76-Y88, Fig. S1) and prevents interaction with IL-12Rβ1 (18). These data suggest IL-12 and IL-23 bind to IL-12Rβ1 *via* p40. Interestingly, the binding area of the anti-human p40 22E11 nanobody overlaps with the one of Ustekinumab (19). In 2015, we showed that deletion of D1 in murine p40 prevents interaction of p40D2D3/p19 with murine IL-12Rβ1 suggesting p40 overtakes the site 2 interaction capacity of p19 (11). Recently, crystal structures of human IL-12Rβ1 and the IL-23 receptor complex (IL-23/IL-23R/IL-12Rβ1) have been reported by Glassman and colleagues (20). In addition, cryo-EM structures of complete IL-12 and IL-23 extracellular domain complexes have been determined (20). Based on these data, Glassman *et al.* hypothesize a single binding interface formed by a contiguous, positively charged loop in p40 (human: H216, K217, and K219) and a hydrophobic strip on p40 (W37 and F82) (Fig. S1). IL-12 and IL-23 variants with multiple alanine mutations (P39A/D40A/E81A/F82A) in two loops of p40 D1 reduce the potency of the cytokines. However, the impact of W37 has not been analyzed (20).

Here, we show that the N-terminal region of p40 constitutes the binding interface to IL-12Rβ1. Furthermore, we confirm W37 of p40 as a mandatory amino acid for site 2 interaction.

## Results

### The amino acids Y31 to T38 of murine p40 are critically involved in binding of murine IL-23 to murine IL-12Rβ1

Considering that (i) the N-terminal region of gp130 is required for formation of the IL-6 receptor complex (21), and (ii) Ustekinumab neutralizes the activity of IL-12 and IL-23 *via* binding to D1 of p40 (18), we suggest that the N-terminal region of p40 is involved in binding to IL-12Rβ1. In order to prove our hypothesis, we generated four N-terminal deletion variants of murine p40 (mp40) in murine Hyper-IL-23 (HIL-23). HIL-23 is a fusion protein of mp40 and murine p19 (mp19) connected by a flexible peptide linker (22, 23). This strategy largely prevents the formation of free p40 homodimers, which may have antagonistic properties in our assays as reported previously (15). Each of the four HIL-23 deletion variants (ΔM23-D29, ΔM23-E34, ΔM23-P39, and ΔY31-T38, Fig. S1) contains an Ig κ chain signal peptide and an N-terminal FLAG tag.

**Figure 1:**
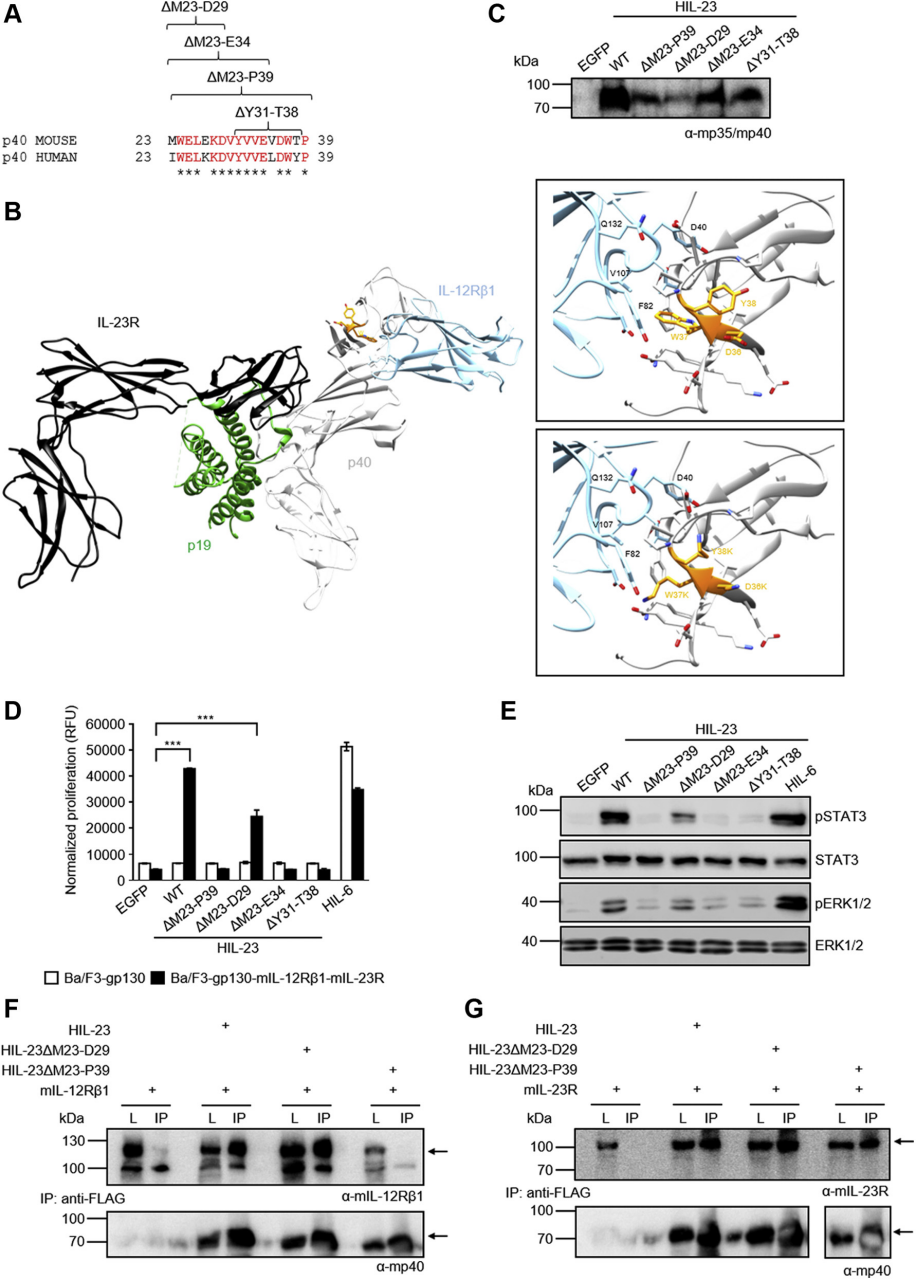
**The N-terminal region of murine p40 from Y31 to T38 mediates binding to murine IL-12Rβ1.***A*, alignment of murine and human p40 N-terminal amino acids with analyzed mp40 deletion variants. *B*, crystal structure of the IL-23:IL-23R:IL-12Rβ1 complex (PDB 6WDQ). *Insets* show zoomed in views on the area surrounding residues D36-Y38. D36-Y38 are depicted in *orange stick* representation. p40 and IL-12Rβ1 residues in contact with D36-Y38 are displayed in *stick* representation. p40 mutations D36K, W37K and Y38K were visualized in the *second inset*. *C*, Western blot analysis of secreted murine HIL-23 variants from transfected CHO-K1 cells. *D*, cellular proliferation of Ba/F3-gp130-mIL-12Rβ1-mIL-23R cells. The cells were cultured for 3 days in the presence of 10 ng/ml HIL-6 or with the indicated cytokines (10% conditioned cell culture supernatant of transfected CHO-K1 cells). Parental Ba/F3-gp130 cells were used as controls. The results of one representative experiment of four are shown. Error bars represent S.D. for technical replicates. Statistical analysis used a one-way ANOVA, followed by Bonferroni correction (n = 3), ∗∗∗*p* ≤ 0.001. *E*, analysis of STAT3 and ERK1/2 activation. Ba/F3-gp130-mIL-12Rβ1-mIL-23R cells were washed, starved, and stimulated with the indicated cytokines (10% conditioned cell culture supernatant of transfected CHO-K1 cells) for 30 min. Cellular lysates were prepared, and equal amounts of total protein (50 μg/lane) were loaded on SDS-PAA gels, followed by immunoblotting using specific antibodies for phospho-STAT3, STAT3, phospho-ERK1/2, and ERK1/2. Western blotting data show results of one representative experiment of two. *F*, co-IP of FLAG-tagged murine HIL-23 variants (wild-type, ΔM23-D29, ΔM23-P39) and full-length mIL-12Rβ1. The position of mIL-12Rβ1 and HIL-23 variants is indicated by *arrows*. One of two independent experiments is shown. *G*, co-IP of FLAG-tagged murine HIL-23 variants (wild-type, ΔM23-D29, ΔM23-P39) and full-length mIL-23R. The position of mIL-23R and HIL-23 variants is indicated by *arrows*. One of two independent experiments is shown.

Alignment of the first 17 amino acids of secreted p40 shows 76.5% homology between mouse and men, while the overall homology of p40 is about 66.7% (Fig. 1*A*). The structure of IL-23 reveals that the N-terminus of p40 is freely accessible for interaction with IL-12Rβ1 (Fig. 1*B*). p40 displays extensive domain flexibility manifested by a hinge-like motion of D1 or D3 of 5° to 10° with respect to D2 (12).

Proliferation of murine Ba/F3 cells stably transduced with gp130, murine IL-23R, and murine IL-12Rβ1 depends on HIL-23 or Hyper-IL-6 (HIL-6) (23). Cell culture supernatants of CHO-K1 cells secreting HIL-23 or deletion variants thereof (Fig. 1*C*) were used to stimulate Ba/F3-gp130-mIL-12Rβ1-mIL-23R cells. Proliferation was induced by HIL-23ΔM23-D29 but not by the other three deletion variants (Fig. 1*D*). In total, 10% of conditioned cell culture supernatants from CHO-K1 cells have been used for cell stimulation to show activity of cytokines with lower secretion levels. Dose–response studies of Ba/F3-gp130-mIL-12Rβ1-mIL-23R cells revealed that saturation of HIL-23-dependent cell proliferation is achieved with 1% of supernatant (Fig. S2). Analysis of STAT3 and ERK1/2 phosphorylation, which are part of the intracellular signal transducing machinery, showed that only HIL-23ΔM23-D29 was able to initiate signaling (Fig. 1*E*), suggesting that the amino acids from Y31 to T38 are critical for signal transduction. To investigate whether binding of HIL-23 to mIL-12Rβ1 is lost in the mutant version, cytokine/cytokine receptor immunoprecipitation studies with two deletion variants were performed. As a control, both cytokine variants should still bind mIL-23R. For coimmunoprecipitation studies, lysates with heterologous expressed murine IL-12Rβ1 (mIL-12Rβ1) and HIL-23 or variants thereof were combined. Precipitation of HIL-23 using FLAG mAbs resulted in coimmunoprecipitation of mIL-12Rβ1 and mIL-23R (Fig. 1, *F* and *G*). While both HIL-23ΔM23-D29 and HIL-23ΔM23-P39 interacted with mIL-23R (Fig. 1*G*), interaction with mIL-12Rβ1 was only seen for HIL-23ΔM23-D29 and HIL-23 (Fig. 1*F*). Taken together, our data show that the N-terminal region of p40 from Y31 to T38 mediates binding to mIL-12Rβ1.

### The amino acid W37 is critically involved in binding of murine IL-23 to murine IL-12Rβ1

**Figure 2:**
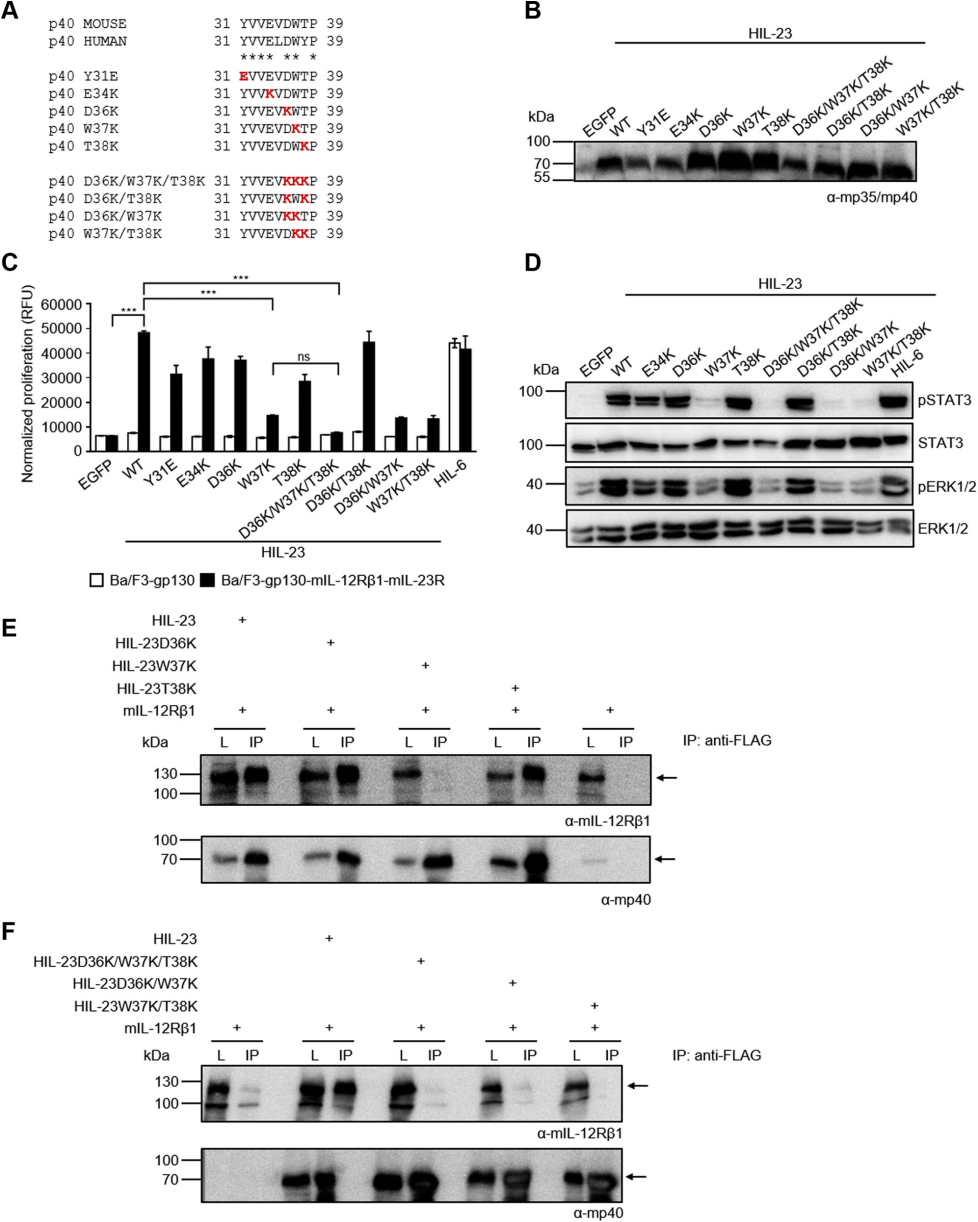
**W37 of murine p40 is important for binding of HIL-23 to murine IL-12Rβ1.***A*, alignment of murine and human p40 N-terminal amino acids Y31 to P39. Single, double, and triple substitutions within mp40 are highlighted in *red*. *B*, Western blot analysis of secreted murine HIL-23 variants from transfected CHO-K1 cells. *C*, cellular proliferation of Ba/F3-gp130-mIL-12Rβ1-mIL-23R cells. The cells were cultured for 3 days in the presence of 10 ng/ml HIL-6 or with the indicated cytokines (10% conditioned cell culture supernatant of transfected CHO-K1 cells). Parental Ba/F3-gp130 cells were used as controls. The results of one representative experiment of three are shown. Error bars represent S.D. for technical replicates. Statistical analysis used a one-way ANOVA, followed by Bonferroni correction (n = 3), ∗∗∗*p* ≤ 0.001, ns not significant. *D*, analysis of STAT3 and ERK1/2 activation. Ba/F3-gp130-mIL-12Rβ1-mIL-23R cells were washed, starved, and stimulated with the indicated cytokines (10% conditioned cell culture supernatant of transfected CHO-K1 cells) for 30 min. Cellular lysates were prepared, and equal amounts of total protein (50 μg/lane) were loaded on SDS-PAA gels, followed by immunoblotting using specific antibodies for phospho-STAT3, STAT3, phospho-ERK1/2, and ERK1/2. Western blotting data show results of one representative experiment of three. *E*, co-IP of FLAG-tagged murine HIL-23 variants (wild-type, D36K, W37K and T38K) and full-length mIL-12Rβ1. The position of mIL-12Rβ1 and HIL-23 variants is indicated by *arrows*. One of two independent experiments is shown. *F*, co-IP of FLAG-tagged murine HIL-23 variants (wild-type, D36K/W37K/T38K, D36K/W37K, W37K/T38K) and full-length mIL-12Rβ1. The position of mIL-12Rβ1 and HIL-23 variants is indicated by *arrows*. One of two independent experiments is shown.

Single hotspot amino acids within the amino acid sequence Y31 to T38 of p40 may contribute to IL-12Rβ1 binding. Consequently, the following p40 point mutations were introduced in HIL-23: Y31E, E34K, D36K, W37K, T38K (Fig. 2*A*). Recently, the aromatic residue W37 was proposed as hotspot amino acid for interaction of p40 with IL-12Rβ1 (20). W37 is at the center of a hydrophobic interface formed by residues V107, L108, D101, and Y134 of the IL-12Rβ1 as well as F82 of p40 (Fig. 1*B*) (20). In general, we introduced the positively charged amino acid lysine (K) to replace negatively charged (E34, D36), polar (T38), or hydrophobic (W37) amino acids except for Y31, where we replaced the polar amino acid Y into a negatively charged glutamic acid (E). Our aim was to achieve strong repulsion by introduction of a large, positively charged amino acid. Additionally, we generated three double (D36K/T38K; D36K/W37K; W37K/T38K) and one triple mutant (D36K/W37K/T38K, KKK) (Fig. 2*A*). Ba/F3-gp130-mIL-12Rβ1-mIL-23R cells were stimulated with conditioned cell culture supernatants of CHO-K1 cells containing the HIL-23 mutation variants (Fig. 2*B*), and proliferation was quantified. The single mutant HIL-23W37K diminished cellular proliferation (Fig. 2*C*). Analysis of STAT3 and ERK1/2 phosphorylation confirmed reduced signaling for HIL-23W37K (Fig. 2*D*). Single mutation of p40 T38 or D36 has no influence on IL-23 activity. Changing D36/W37/T38 into D36K/W37K or W37K/T38K was comparable to the single mutation HIL-23W37K. Interestingly, substitution of all three amino acids into KKK completely prevented induction of cellular proliferation and STAT3/ERK1/2 phosphorylation by HIL-23 (Fig. 2, *C* and *D*).

Secondary structure analysis by circular dichroism (CD) spectroscopy ruled out the possibility that observed effects are due to a possible improper folding of the KKK mutant compared with wild-type.

Far-UV CD spectroscopy analysis of purified HIL-23 and HIL-23D36K/W37K/T38K (Figs. S3 and S4) indicated a mixed α-helical/β-sheet structure characterized by a minimum of 209.4 nm (HIL-23) or 211.2 nm (HIL-23D36K/W37K/T38K) and an x-axis intercept of 200.5 nm (HIL-23) or 201.3 nm (HIL-23D36K/W37K/T38K), respectively, which is in good agreement with its crystal structure (Figs. 1*B* and S4, *A* and *B*). The overall secondary structure of HIL-23D36K/W37K/T38K is highly similar to the overall secondary structure of HIL-23 (Fig. S4) and indicates that the KKK mutation does not alter the overall structure of the protein. Furthermore, receptor-binding assays by surface plasmon resonance spectroscopy (SPR) revealed binding of HIL-23D36K/W37K/T38K to the IL-23R with comparable affinity to HIL-23 (Fig. S5 and Table 1).

**Table 1:** Kinetic parameters of the interaction between the cytokine and the respective receptor

Cytokine	Receptor	k_on_ (M^−1^ s^−1^)	k_off_ (s^−1^)	K_D_ (nM)
HIL-23	IL-23R	3.0 × 10^4^	1.2 × 10^−3^	40
HIL-23D36K/W37K/T38K	IL-23R	1.9 × 10^4^	1.1 × 10^−3^	57
HIL-12	IL-12Rβ2	1.9 × 10^6^	1.9 × 10^−3^	0.987
HIL-12D36K/W37K/T38K	IL-12Rβ2	3.2 × 10^6^	2.7 × 10^−3^	0.840

SPR analysis of HIL-23 and HIL-12 variants binding to IL-23R-Fc and IL-12Rβ2-Fc. IL-23R-Fc and IL-12Rβ2-Fc were immobilized on a Protein A chip and increasing concentrations of HIL-23 or HIL-12 variants were injected at a flow rate of 30 μl/min. Measurements were carried out on a Biacore X100 instrument.

A possible explanation for reduced binding found for the KKK variant to IL-12Rβ1 may be T38K salt bridge formation with D40 (Fig. 1*B*). This may result in weakening the interaction of p40 to IL-12Rβ1 through loss of D40 interaction with IL-12Rβ1 Q132. D36K mutation will likely result in repulsion of T38K and D36K further increasing the likelihood of T38K interaction with D40. Despite binding to IL-23R (Fig. S6, *A* and *B*), introduction of W37K in HIL-23W37K, HIL-23D36K/W37K, HIL-23W37K/T38K, and HIL-23D36K/W37K/T38K resulted in abrogated binding to IL-12Rβ1 (Fig. 2, *E* and *F*).

**Figure 3:**
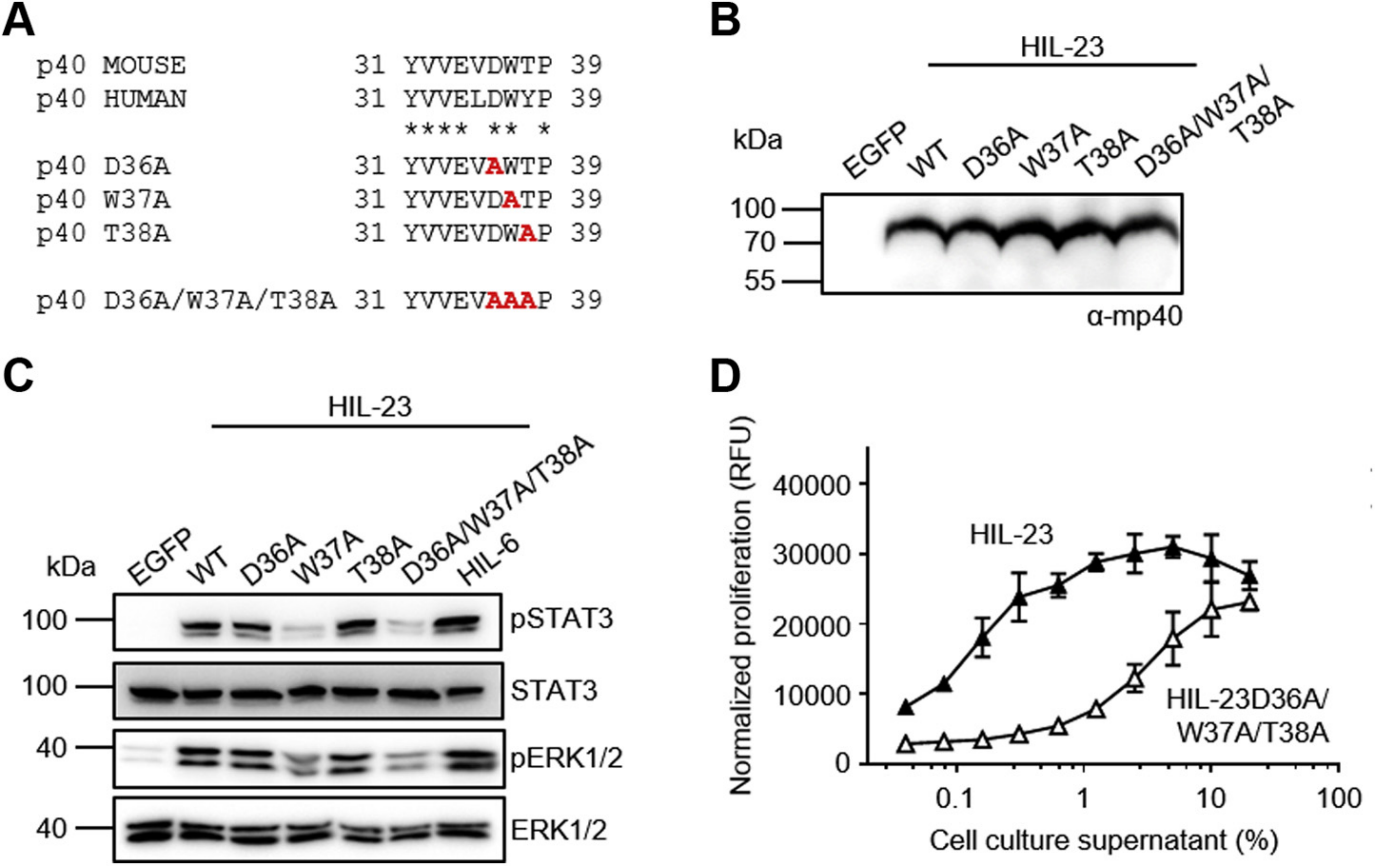
**Alanine substitutions of p40 D36, W37 and T38 impaired HIL-23 activity.***A*, alignment of murine and human p40 N-terminal amino acids Y31 to P39. Single and triple substitutions within mp40 are highlighted in *red*. *B*, Western blot analysis of secreted murine HIL-23 variants from transfected CHO-K1 cells. Cytokine variants were transiently expressed with comparable efficiency. *C*, analysis of STAT3 and ERK1/2 activation. Ba/F3-gp130-mIL-12Rβ1-mIL-23R cells were washed, starved, and stimulated with the indicated cytokines (10% conditioned cell culture supernatant of transfected CHO-K1 cells) for 30 min. Cellular lysates were prepared, and equal amounts of total protein (50 μg/lane) were loaded on SDS-PAA gels, followed by immunoblotting using specific antibodies for phospho-STAT3, STAT3, phospho-ERK1/2, and ERK1/2. Western blotting data show results of one representative experiment of three. *D*, cellular proliferation of Ba/F3-gp130-mIL-12Rβ1-mIL-23R cells. The cells were cultured for 3 days in the presence of the indicated cytokines (0.04–20% conditioned cell culture supernatant of transfected CHO-K1 cells). The results of one representative experiment of four are shown. Error bars represent S.D. for technical replicates.

To ensure the importance of p40W37 in binding of IL-23 to the IL-12Rβ1, we introduced alanine mutations at position D36, W37, or T38 and expressed the resulting HIL-23 variants in CHO-K1 cells (Fig. 3, *A* and *B*). Phosphorylation of STAT3 and ERK1/2 was tremendously reduced in Ba/F3-gp130 cells expressing mIL-23R and mIL-12Rβ1 upon stimulation with HIL-23W37A and HIL-23D36A/W37A/T38A (AAA, Fig. 3*C*). Proliferation of Ba/F3-gp130-mIL-12Rβ1-mIL-23R cells induced by HIL-23 was strongly reduced by the triple mutation D36A/W37A/T38A compared with wild-type HIL-23 (Fig. 3*D*). W37A mutation leads to a loss of the hydrophobic interactions formed with V107, L108, and Y134 of IL-12Rβ1 and thus efficiently diminishes the biological activity of the AAA variant. This further highlights that W37 of p40 is mandatory for binding of IL-23 to IL-12Rβ1.

**Figure 4:**
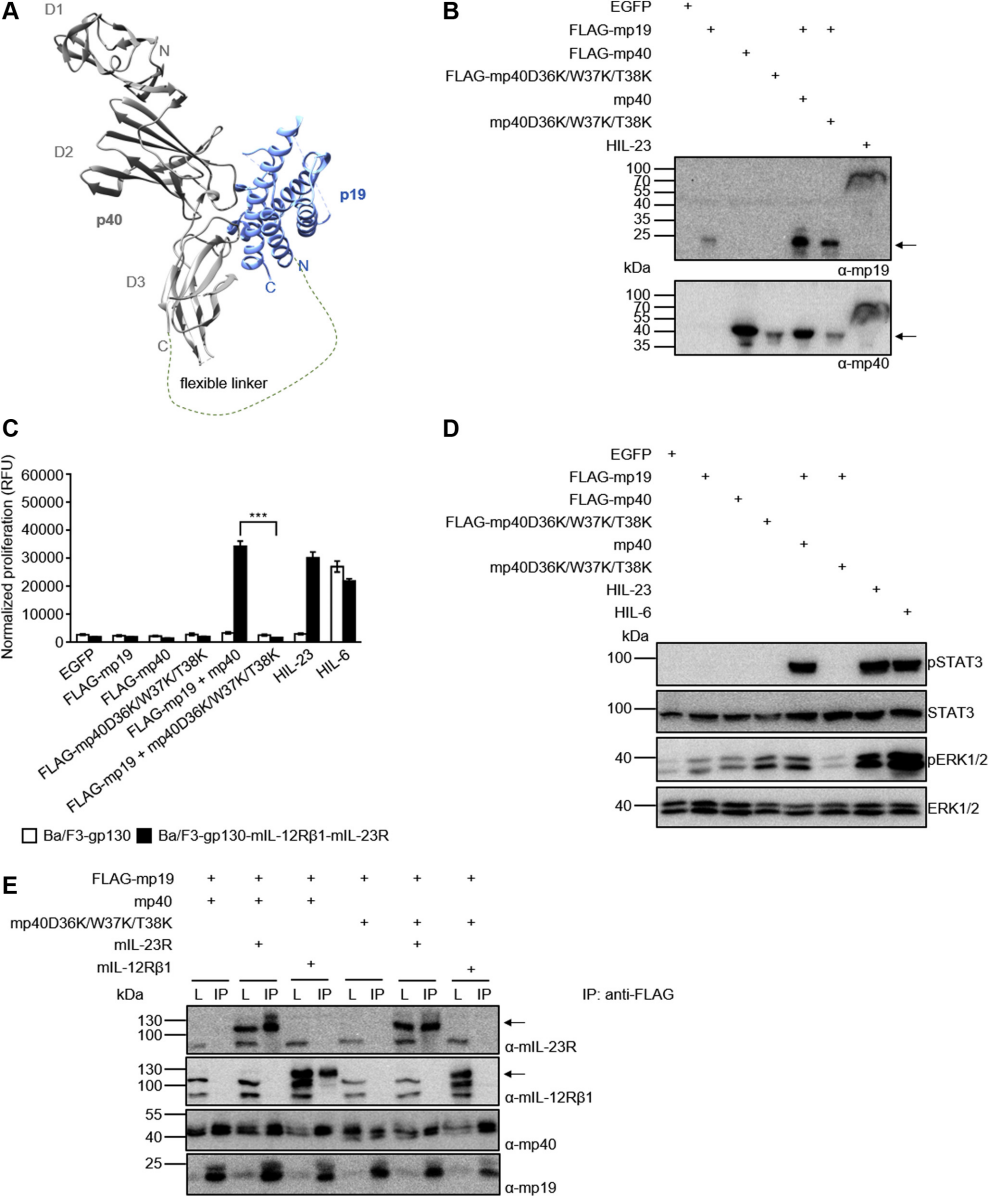
**Murine p40 with D36K/W37K/T38K substitutions interacts with murine p19 but not with murine IL-12Rβ1.***A*, structure of hIL-23 extracted from the structure of the IL-23:IL-23R complex structure (PDB 5mzv). A flexible linker sequence connecting p40 and p19 is indicated. *B*, Western blot analysis of secreted murine HIL-23, mp19 and mp40 variants from transfected CHO-K1 cells. The position of mp19 and mp40 variants is indicated by *arrows*. *C*, cellular proliferation of Ba/F3-gp130-mIL-12Rβ1-mIL-23R cells. The cells were cultured for 3 days in the presence of 10 ng/ml HIL-6 or with the indicated cytokines (10% conditioned cell culture supernatant of transfected CHO-K1 cells). Parental Ba/F3-gp130 cells were used as controls. The results of one representative experiment of three are shown. Error bars represent S.D. for technical replicates. Statistical analysis used a one-way ANOVA, followed by Bonferroni correction (n = 3), ∗∗∗*p* ≤ 0.001. *D*, analysis of STAT3 and ERK1/2 activation. Ba/F3-gp130-mIL-12Rβ1-mIL-23R cells were washed, starved, and stimulated with the indicated cytokines (10% conditioned cell culture supernatant of transfected CHO-K1 cells) for 30 min. Cellular lysates were prepared, and equal amounts of total protein (50 μg/lane) were loaded on SDS-PAA gels, followed by immunoblotting using specific antibodies for phospho-STAT3, STAT3, phospho-ERK1/2, and ERK1/2. Western blotting data show results of one representative experiment of two. *E*, co-IP of FLAG-tagged murine mp19 coexpressed with either mp40 or mp40D36K/W37K/T38K and full-length mIL-12Rβ1 or mIL-23R. The position of mIL-23R and mIL-12Rβ1 is indicated by *arrows*. One of two independent experiments is shown.

Generally, we used HIL-23 fusion proteins for our analyses (Fig. 4*A*). However, to demonstrate the importance of p40D36/W37/T38 for binding of natural IL-23 to IL-12Rβ1, we cotransfected p19 and p40 or p40D36K/W37K/T38K, respectively, into CHO-K1 cells (Fig. 4*B*). As anticipated, only p19/p40 induced cellular proliferation and STAT3/ERK1/2 phosphorylation of Ba/F3-gp130-mIL-12Rβ1-mIL-23R cells (Fig. 4, *C* and *D*). Coimmunoprecipitation studies showed p19/p40 interaction with both receptors, whereas p19/p40D36K/W37K/T38K bound to IL-23R but not to IL-12Rβ1 (Fig. 4*E*).

Summing up, we confirmed that the aromatic residue W37 is indispensable for p40 binding to mIL-12Rβ1.

### The amino acid sequence D36/W37/T38 is critically involved in binding of murine IL-12 to murine IL-12Rβ1

**Figure 5:**
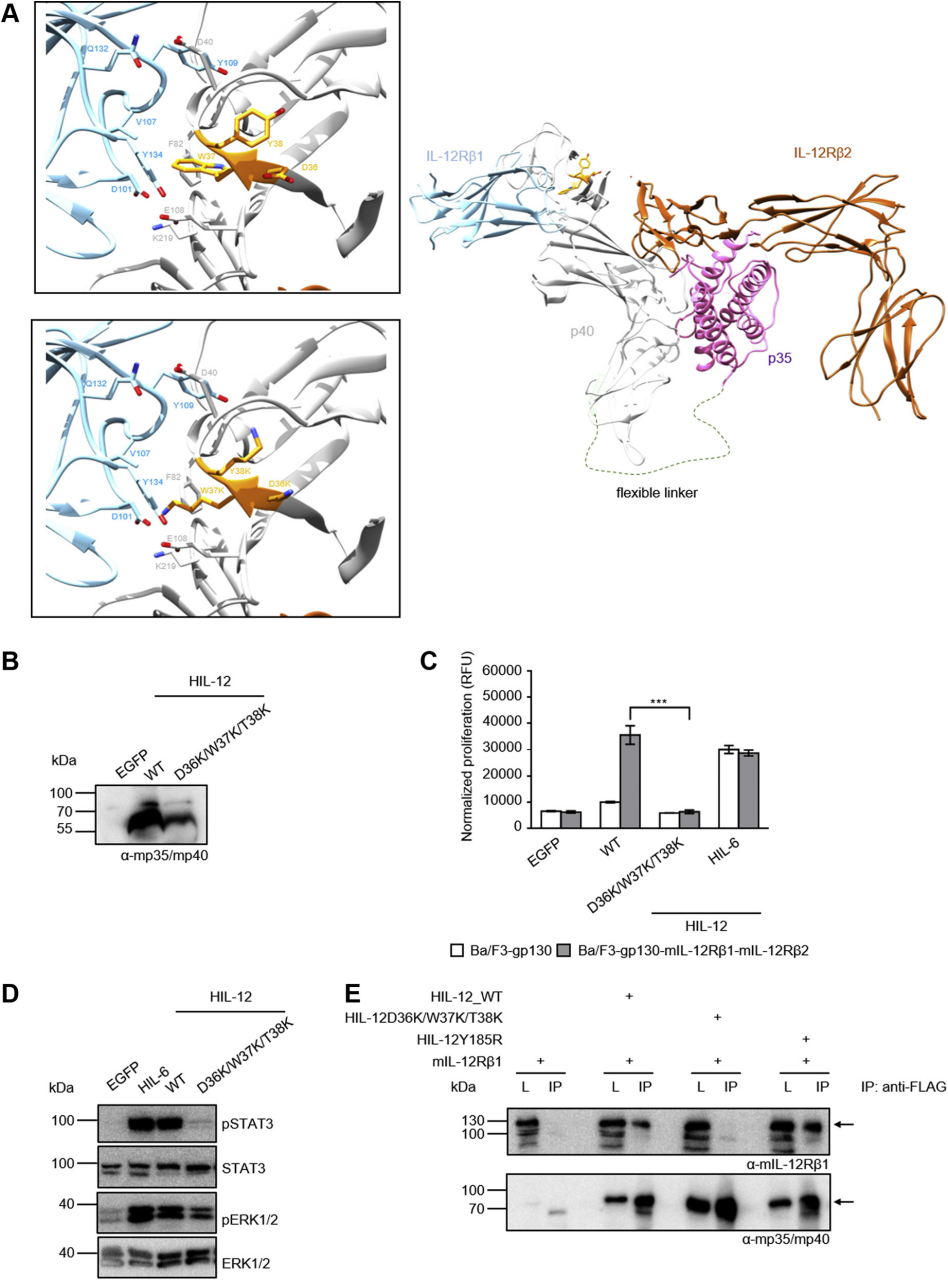
**Amino acid sequence D36/W37/T38 in murine p40 is critically involved in binding of murine IL-12 to IL-12Rβ1.***A*, model of the IL-12 signaling complex. A model of IL-12 (PDB 3HMX) in complex with models of IL-12Rβ1 and IL-12Rβ2 was generated based on the architecture of the IL-23:IL-23R:IL-12Rβ1 crystal structure (PDB 6WDQ). *Insets* show a zoomed in view on the area surrounding residues D36-Y38. Critical binding interface residues D36-Y38 of p40 are depicted in *orange stick* representation, IL-12Rβ1 and p40 residues in contact with D36-Y38 are displayed in *stick* representation. p40 mutations D36K, W37K, and Y38K were visualized in the *second inset*. *B*, Western blot analysis of secreted murine HIL-12 variants (wild-type, D36K/W37K/T38K) from transfected CHO-K1 cells. *C*, cellular proliferation of Ba/F3-gp130-mIL-12Rβ1-mIL-12Rβ2 cells. The cells were cultured for 3 days in the presence of 10 ng/ml HIL-6 or with the indicated cytokines (10% conditioned cell culture supernatant of transfected CHO-K1 cells). Parental Ba/F3-gp130 cells were used as controls. The results of one representative experiment of three are shown. Error bars represent S.D. for technical replicates. Statistical analysis used a one-way ANOVA, followed by Bonferroni correction (n = 3), ∗∗∗*p* ≤ 0.001. *D*, analysis of STAT3 and ERK1/2 activation. Ba/F3-gp130-mIL-12Rβ1-mIL-12Rβ2 cells were washed, starved, and stimulated with the indicated cytokines (10% conditioned cell culture supernatant of transfected CHO-K1 cells) for 30 min. Cellular lysates were prepared, and equal amounts of total protein (50 μg/lane) were loaded on SDS-PAA gels, followed by immunoblotting using specific antibodies for phospho-STAT3, STAT3, phospho-ERK1/2, and ERK1/2. Western blotting data show results of one representative experiment of three. *E*, co-IP of FLAG-tagged murine HIL-12 variants (wild-type, D36K/W37K/T38K, Y185R) and full-length mIL-12Rβ1. The position of mIL-12Rβ1 and HIL-12 variants is indicated by *arrows*. One of two independent experiments is shown.

IL-12 and IL-23 both interact with the IL-12Rβ1 *via* the p40 subunit, whereas p35 interacts with IL-12Rβ2 and p19 with the IL-23R. In order to test whether p40D36/W37/T38 is required for binding of IL-12 to IL-12Rβ1, we introduced D36K/W37K/T38K into Hyper-IL-12 (HIL-12) (Fig. 5*A*) and expressed the proteins in CHO-K1 cells (Fig. 5*B*). Analogous to inactivating mutations introduced into HIL-23, we performed experiments to ensure proper folding and receptor binding *via* CD spectroscopy and SPR (Figs. S4 and S5). Far-UV CD spectra were characterized by a minimum of 209.4 nm (HIL-12) or 209.6 nm (HIL-12D36K/W37K/T38K) and an x-axis intercept of 200.9 nm (HIL-12) or 200.1 nm (HIL-12D36K/W37K/T38K), showing a mixed α-helical/β-sheet structure in good agreement with the crystal structure of HIL-12 (Figs. 5*A* and S4, *C* and *D*). Like in case of HIL-23D36K/W37K/T38K, HIL-12D36K/W37K/T38K retained proper folding and demonstrated binding to IL-12Rβ2 comparable to HIL-12 (Table 1 and Fig. S5).

We showed previously that IL-12 selectively induced activation of STAT1 and STAT3 in Ba/F3 cells (24), because Ba/F3 cells have very low STAT4 protein levels and cannot be used for analysis of IL-12-induced STAT4 activation (25). HIL-12D36K/W37K/T38K was biologically inactive on Ba/F3-gp130-mIL-12Rβ1-mIL-12Rβ2 cells (Fig. 5, *C* and *D*). Coimmunoprecipitation revealed interaction of HIL-12D36K/W37K/T38K with IL-12Rβ2 but not with IL-12Rβ1, whereas HIL-12 precipitated both IL-12Rβ1 and IL-12Rβ2 (Figs. 5*E* and S6*C*). As a control, we used site 3 variant HIL-12Y185R, which interacts with IL-12Rβ1 and not IL-12Rβ2 (13). In summary, our data indicate that D36/W37/T38 is mandatory in p40 for interaction with IL-12Rβ1.

### The amino acid sequence D36/W37/T38 is important for binding of homodimeric p40 to IL-12Rβ1

**Figure 6:**
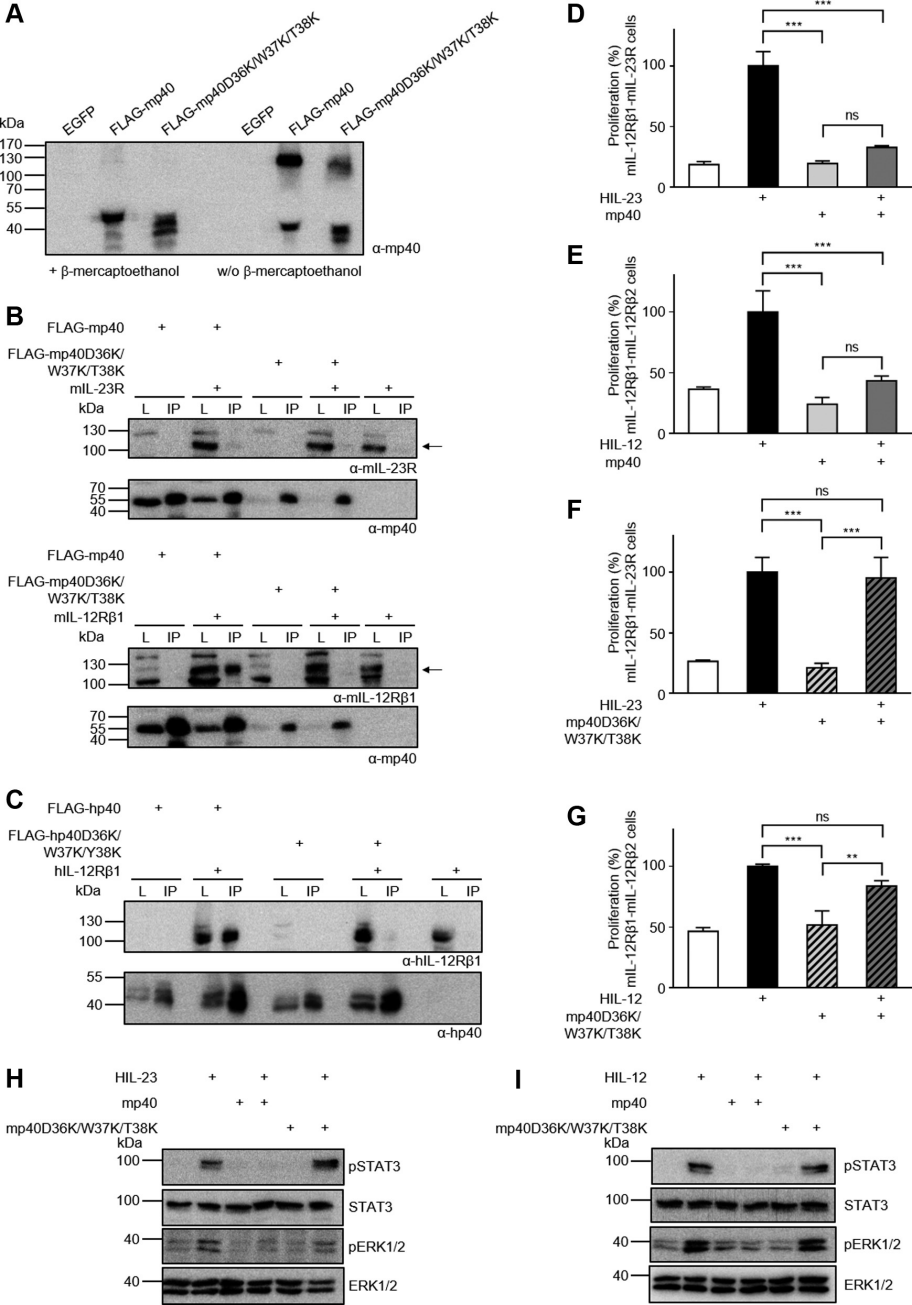
**Substitution of the amino acid sequence D36/W37/T38 diminished antagonistic properties of homodimeric p40.***A*, Western blot analysis of secreted mp40 and mp40D36K/W37K/T38K conditions. In total, 20 μl conditioned supernatant of transiently transfected CHO-K1 was separated by SDS-PAGE under (non-)reducing conditions, and proteins were visualized by Western blotting with a mp40-specific antibody. *B*, co-IP of FLAG-tagged murine p40 variants (wild-type, D36K/W37K/T38K) and full-length mIL-23R or mIL-12Rβ1. The position of mIL-23R and mIL-12Rβ1 is indicated by *arrows*. One of two independent experiments is shown. *C*, co-IP of FLAG-tagged human p40 variants (wild-type, D36K/W37K/Y38K) and full-length hIL-12Rβ1. One of two independent experiments is shown. *D*, cellular proliferation of Ba/F3-gp130-mIL-12Rβ1-mIL-23R cells. The cells were cultured for 3 days in the presence of 7.5 ng/ml murine HIL-23 with or without 2 μg/ml mp40. The results of one representative experiment of two are shown. Error bars represent S.D. for technical replicates. Statistical analysis used a one-way ANOVA, followed by Bonferroni correction (n = 3), ∗∗∗*p* ≤ 0.001, ns not significant. *E*, cellular proliferation of Ba/F3-gp130-mIL-12Rβ1-mIL-12Rβ2 cells. The cells were cultured for 3 days in the presence of 5 ng/ml murine HIL-12 with or without 2 μg/ml mp40. The results of one representative experiment of three are shown. Error bars represent S.D. for technical replicates. Statistical analysis used a one-way ANOVA, followed by Bonferroni correction (n = 3), ∗∗∗*p* ≤ 0.001, ns not significant. *F*, cellular proliferation of Ba/F3-gp130-mIL-12Rβ1-mIL-23R cells. The cells were cultured for 3 days in the presence of 7.5 ng/ml murine HIL-23 with or without 2 μg/ml mp40D36K/W37K/T38K. The results of one representative experiment of two are shown. Error bars represent S.D. for technical replicates. Statistical analysis used a one-way ANOVA, followed by Bonferroni correction (n = 3), ∗∗∗*p* ≤ 0.001, ns not significant. *G*, cellular proliferation of Ba/F3-gp130-mIL-12Rβ1-mIL-12Rβ2 cells. The cells were cultured for 3 days in the presence of 5 ng/ml murine HIL-12 with or without 2 μg/ml mp40D36K/W37K/T38K. The results of one representative experiment of three are shown. Error bars represent S.D. for technical replicates. Statistical analysis used a one-way ANOVA, followed by Bonferroni correction (n = 3), ∗∗∗*p* ≤ 0.001, ∗∗*p* ≤ 0.01, ns not significant. *H* and *I*, analysis of STAT3 and ERK1/2 activation. Ba/F3-gp130-mIL-12Rβ1-mIL-23R or Ba/F3-gp130-mIL-12Rβ1-mIL-12Rβ2 cells were washed and starved for at least 4 h. One hour prior to stimulation 2 μg/ml mp40 or mp40D36K/W37K/T38K have been added. Cells were stimulated with the indicated cytokines (7.5 ng/ml HIL-23, 5 ng/ml HIL-12) for 30 min. Cellular lysates were prepared, and equal amounts of total protein (50 μg/lane) were loaded on SDS-PAA gels, followed by immunoblotting using specific antibodies for phospho-STAT3, STAT3, phospho-ERK1/2, and ERK1/2. Western blotting data show results of one representative experiment of two.

If expressed without p19 or p35, p40 forms the covalently linked p40 homodimer p80, which interacts with IL-12Rβ1 to antagonize IL-12 and IL-23. We expressed mp40 and mp40D36K/W37K/T38K in CHO-K1 cells and showed formation of p80 by (non-)reducing SDS-PAGE followed by detection of p40 (Fig. 6*A*). mp40D36K/W37K/T38K failed to interact with mIL-12Rβ1 and neither mp40 nor mp40D36K/W37K/T38K bound to mIL-23R (Fig. 6*B*). These data were confirmed for human p40 (hp40) variants and their binding to human IL-12Rβ1 and human IL-23R (Figs. 6*C* and S6*D*). The homology of murine and human p40 is visualized in Fig. S1.

HIL-12 and HIL-23-induced proliferation and STAT3/ERK1/2 phosphorylation of respective Ba/F3-gp130 cells were inhibited by recombinant mp40 but not by mp40D36K/W37K/T38K (Figs. 6, *D*–*I* and S3).

Irrespective of p40 being present in IL-12, IL-23, or as homodimeric p40 (p80), our data suggest that the binding mode of p40 to IL-12Rβ1 is identical for all three proteins and strongly depends on the N-terminal amino acids D36/W37/T38.

## Discussion

IL-12 and IL-23 are the key cytokines of the IL-12-type cytokine family and share main structural signatures. Both cytokines are composed of the β subunit p40, which mediates binding to the common IL-12Rβ1. The specific receptors IL-12Rβ2 and IL-23R bind to the α subunits p35 and p19, respectively (1). Interaction of p19/p35 and p40 is mediated by domains 2 and 3 of p40 indicating that these domains are dispensable for IL-12Rβ1 interaction. Recently, crystal structure of the IL-23 receptor complex and cryo-EM maps of the complete IL-12 and IL-23 receptor complexes have been published (20). The shared p40 subunit directly interacts with IL-12Rβ1 indicating a convergent function of p40 in mediating the assembly of IL-12 and IL-23 signaling complexes (20). The closely related IL-6 is a composite cytokine, which must interact with IL-6R before binding to gp130. In contrast to p40, IL-6R does not contribute to binding to gp130. However, the N-terminal region of gp130 is mandatory for interaction with IL-6 (21). Consequently, we analyzed the requirement of the N-terminus of p40 for binding to IL-12Rβ1. Deletion of the first seven N-terminal amino acids (M23–D29) had no influence on the biological activity of HIL-23. However, deletion of adjacent p40 amino acids Y31 to T38 completely abolished IL-23 biological activity and binding to IL-12Rβ1. This finding aligns with the structural data from Glassman and colleagues, where they hypothesized that the p40/IL-12Rβ1 interface is formed by a charged loop and a hydrophobic strip on p40 due to the aromatic residues W37 and F82 (20). Furthermore, by deletion of amino acids Y31 to T38, we partially disrupted loop 1 in domain 1 of p40, which is important for binding of the IL-12/IL-23 cross-neutralizing monoclonal antibody Ustekinumab (18). Structural mapping of the p40 epitope of Ustekinumab showed interaction with some surface-exposed residues within the first three loops W37-M45, L62-L69, T76-Y88 of p40 domain 1. W37 was found to be the critical amino acid for the interaction of Ustekinumab with p40 (18). Based on these findings, we are able to pinpoint W37 and the surrounding amino acids D36 and T38 as hotspot amino acids for interaction with IL-12Rβ1 *via* site 2. IL-12/IL-23 signaling and binding of p40 to IL-12Rβ1 were diminished for p40W37K and completely abrogated for p40D36K/W37K/T38K. These amino acids are almost identical between mice (DWT) and men (DWY). Consequently, binding of murine and human p40 to IL-12Rβ1 is largely identical in this area. For Ustekinumab, which binds to human but not to murine p40, at least 18 amino acids of p40 domain 1 mediate the binding interface (W37; D40; P42; G43; M45; L62; Q64; S65; E67; L69; I77; Q78; K80; E81; F82; G83; D84; Y88) (18). Amino acids of loop 2 (L62-L69) are largely different between mice and men (Fig. S1*B*), which may explain why Ustekinumab does not interact with murine p40. Mutational analysis showed that in particular M45, L62, S65, E67, E81, and D84 of the domain 1 are important residues within the binding epitope to Ustekinumab (18). In these studies, W37 has not been analyzed. This does, however, not necessarily mean that these amino acids (M45, L62, S65, E67, E81, and D84) are most critical for interaction with IL-12Rβ1, because Ustekinumab may simply bind close to the p40:IL-12Rβ1 interface and may block interaction by structural interference. Interestingly, the anti-human IL-23/IL-12 nanobody 22E11 interacts with the N-terminal domain of human p40 and overlaps with the binding epitope of Ustekinumab (19). We confirmed the hypothesized aromatic W37 within p40 as a hotspot amino acid for interaction of p40 and IL-12Rβ1 (20). In our studies, we did not mutate any other amino acids, which might be relevant for the interaction of IL-12Rβ1 and p40. The goal of our study was to mutate one critical hotspot amino acid stretch to destroy interaction between p40 and IL-12Rβ1 without hindering the interaction of p40:p40, p40:p19; p40:35 and the interaction of IL-12 and IL-23 with IL-12Rβ2 and IL-23R, respectively. We are aware that introduction of amino acid exchanges may simply disrupt the overall configuration/folding within the mutated area and therefore, not directly involved in interaction with IL-12Rβ1. However, we have good evidence supported by recently published structural data that the W37K variant switches the binding interface from binding into nonbinding mode. We show that p40 deletion and exchange variants are still secreted, soluble, correctly folded, and active in a way that enables binding to p19/p35 and, as a consequence, interaction with the second receptor chain IL-23R/IL-12Rβ2. In conclusion, our study provides experimental evidence that W37 of p40 is responsible for direct interaction with IL-12Rβ1 both in IL-12 and IL-23 and in the antagonistic p40 homodimer.

## Experimental procedures

### Cells and reagents

HEK293T (ACC-635) and CHO-K1 cells (ACC-110) were purchased from the Leibniz Institute DSMZ – German Collection of Microorganisms and Cell Cultures. Murine Ba/F3-gp130 cells transduced with mIL-12Rβ1 and mIL-23R, or mIL-12Rβ1 and mIL-12Rβ2 were described previously (23, 24). All cell lines were grown in DMEM high-glucose culture medium (GIBCO, Thermo Fisher Scientific) supplemented with 10% fetal calf serum (GIBCO, Thermo Fisher Scientific), 60 mg/l penicillin, and 100 mg/l streptomycin (Genaxxon bioscience GmbH) at 37 °C with 5% CO_2_ in a water-saturated atmosphere. Proliferation of Ba/F3-gp130 cell lines was maintained by adding 0.2% of conditioned cell culture medium from a stable CHO-K1 clone secreting Hyper-IL-6 (final concentration 10 ng/ml as determined by ELISA). ExpiCHO-S and Expi293F cells were obtained from GIBCO (Thermo Fisher Scientific) and cultured according to manufacturer’s standard protocol. Phospho-STAT3 (Tyr705) (D3A7), STAT3 (124H6), phospho-p44/42 MAPK (ERK1/2) (Thr-202/Tyr-204) (D13.14.4E), and p44/42 MAPK (ERK1/2) antibodies were purchased from Cell Signaling Technology. Peroxidase-conjugated secondary mAbs were obtained from Pierce (Thermo Fisher Scientific). Biotinylated mIL-23R (BAF1686), hIL-23R (BAF1400), mIL-12Rβ1 (BAF1998), hIL-12Rβ1 (BAF839), hIL-12Rβ2 (BAF1959), mp40 (BAF499), mp35/mp40 (BAF419), mp19 (BAF1619), hp40 (BAF219) mAbs, and streptavidin-HRP (DY998) were from R&D Systems. Recombinant mouse IL-23R-Fc chimera protein (1686-MR) and recombinant mouse IL-12Rβ2-Fc chimera protein (7406-MR) were obtained from R&D Systems.

### Cloning

Cloning of pcDNA3.1 expression vectors (Invitrogen) containing an N-terminal FLAG tag and a C-terminal His6 tag for murine Hyper-IL-23 (HIL-23), murine Hyper-IL-12 (HIL-12), murine p40 (FLAG-mp40), and murine p19 (FLAG-mp19) was described elsewhere (11, 23, 24). Standard cloning procedures were used for the generation of pcDNA3.1-mp40 and pcDNA3.1-FLAG-hp40. Mutations within murine/human p40, HIL-23, or HIL-12 were generated by PCR followed by DpnI digestion of methylated template DNA. Expression vectors with Twin-Strep-tag for purification of cytokines have been created by C-terminal insertion of coding sequence for WSHPQFEK-GGGSGGGSGG-SA-WSHPQFEK (iba GmbH). Amino acids are numbered according to the database entry starting with the original signal peptide.

Generation of eukaryotic expression vectors p409 containing the cDNA encoding murine or human IL-23 receptors was described previously (23, 26). Cloning of mIL-12Rβ2 expression vector was described in (24).

### Transfection of cells

CHO-K1 and HEK293T cells (2 × 10^6^) were transiently transfected as indicated using TurboFect transfection reagent (Fermentas, Thermo Scientific) according to the manufacturer’s instructions. ExpiCHO-S and Expi293F cells were transfected according to the manufacturer’s standard protocol (GIBCO, Thermo Fisher Scientific).

### Cell viability assay

To remove the cytokines, Ba/F3-gp130 cell lines were washed three times with sterile PBS. 5 × 10^3^ cells were suspended in DMEM supplemented with 10% FCS, 60 mg/l penicillin, and 100 mg/l streptomycin and cultured for 3 days in a final volume of 100 μl with or without cytokines (applied as conditioned media from transfected CHO-K1 cells) as indicated. The CellTiter-Blue Cell Viability Assay (Promega) was used to estimate viable cells by recording the fluorescence (excitation 560 nm, emission 590 nm) using the Infinite M200 PRO plate reader (Tecan) immediately after adding 20 μl of reagent per well (time point 0) and up to 2 h after incubation under standard cell culture conditions. The fluorescent signal from the CellTiter-Blue Reagent is proportional to the number of viable cells. All values were measured in triplicates per experiment. Fluorescence values were normalized by subtraction of time point 0 values. All experiments were performed at least two times, and one representative experiment was selected. Data are presented as means ± SD. For multiple comparisons, one-way ANOVA, followed by Bonferroni correction, was used (GraphPad Prism 6.0, GraphPad Software Inc). Statistical significance was set at the level of *p* ≤ 0.05 (∗*p* ≤ 0.05, ∗∗*p* ≤ 0.01, ∗∗∗*p* ≤ 0.001).

### Stimulation assays

For analysis of STAT3 and ERK1/2 activation in Ba/F3-gp130 cell lines, cells were starved for at least 4 h in serum-free medium. This was followed by stimulation with cytokines as indicated. Subsequently, cells were harvested and lysed in 10 mM Tris-HCl, pH 7.8, 150 mM NaCl, 0.5 mM EDTA, 0.5% NP-40, 1 mM sodium vanadate, and 10 mM MgCl_2_ supplemented with complete protease inhibitor cocktail tablets (Roche Diagnostics). Protein concentration of cell lysates was determined by BCA protein assay (Pierce, Thermo Scientific) according to the manufacturer’s instructions. Analysis of STAT3 and ERK1/2 activation was done by immunoblotting using 50 μg proteins from total cell lysates and detection with phospho-STAT3 or phospho-ERK1/2 mAbs and STAT3 or ERK1/2 mAbs.

### Coimmunoprecipitation (Co-IP)

For co-IP *via* ANTI-FLAG M2 affinity gel (Sigma Aldrich), transiently transfected HEK293T cells were lysed in 50 mM Tris-HCl pH 7.5, 1 mM EDTA, 150 mM NaCl, and one complete protease inhibitor mixture tablet/50 ml buffer (Roche Diagnostics) supplemented with 1% Triton X-100 for 1 h on ice. Cytokine-containing lysates were mixed with those containing the full-length receptors. For negative control, cytokine variants were incubated without receptors and vice versa. In total, 30 μl of ANTI-FLAG M2 affinity gel (Sigma Aldrich) was added and incubated overnight at 4 °C under gentle agitation. The samples were washed three times with the abovementioned buffer without 1% Triton X-100, and proteins were eluted by adding 50 μl of 2.5× Laemmli buffer, followed by incubation for 10 min at 95 °C. The resulting supernatants were subjected to Western blot analysis.

### Western blotting

In total, 50 μg of proteins from cell lysates or 20 μl of conditioned cell culture supernatants were loaded per lane, separated by SDS-PAGE under reducing conditions, and transferred to PVDF membranes. The membranes were blocked in 5% fat-free dried skimmed milk in TBS-T (10 mM Tris HCl pH 7.6, 150 mM NaCl, 1% Tween 20) and probed with the indicated primary antibodies in 5% fat-free dried skimmed milk in TBS-T (STAT3 1:1000, ERK1/2 1:1000) or 5% BSA in TBS-T (pSTAT3 1:1000, pERK1/2 1:1000, mp40 1:1000, mp19 1:1000, mp35/mp40 1:1000, hp40 1:1000, mIL-23R 1:1000, hIL-23R 1:1000, mIL-12Rβ1 1:300, hIL-12Rβ1 1:300, hIL-12Rβ2 1:300) at 4 °C overnight. After washing, the membranes were incubated with secondary peroxidase-conjugated antibodies (1:5000) or streptavidin-HRP (1:200) diluted in 5% fat-free dried skimmed milk or BSA in TBS-T for 1 h at room temperature. The Immobilon Western Chemiluminescent HRP Substrate (Merck Chemicals GmbH) and the ChemoCam Imager (INTAS Science Imaging Instruments GmbH) were used for signal detection.

### Protein purification

HmIL-12, HmIL-23, and mp40 variants were purified using Strep-TactinXT 4Flow columns according to the manufacturer’s protocol (iba GmbH, Göttingen). Elution was carried out by 50 mM biotin, which specifically competes for the biotin binding pocket. Buffer exchange to PBS was achieved using NAP-25 columns (GE Healthcare). Purified proteins have been analyzed by Coomassie brilliant blue staining.

### Surface plasmon resonance (SPR)

For surface plasmon resonance experiments, a Biacore X100 instrument (Cytiva) was used. Analysis was performed in multi-cycle mode. Experiments were carried out at 25 °C in PBS pH 7.4, composed of 137 mM NaCl, 2.7 mM KCl, 12 mM HPO_4_^2−^ and H_2_PO^4−^, and 0.05% (v/v) surfactant P20 (Cytiva). IL-23R-Fc and IL-12Rβ2-Fc variants were captured on a Protein A chip at a level of ∼150 response units (RUs). HIL-12 or HIL-23 was injected at a flow rate of 30 μl/min at increasing concentrations (0.3–5 nM and 15–1000 nM, respectively). The association in each defined concentration was monitored in periods of 120 s, and the dissociation was measured in periods of 450 s. Final graphs were fitted using a 1:1 binding model.

### Circular dichroism (CD) spectroscopy

Far-UV CD spectra were recorded on a Jasco J-715 spectropolarimeter from 196 to 320 nm with a 0.2 nm step size, 50 nm/min scan speed, and 2 nm bandwidth. Proteins (2.3–4.5 μM) in 0.5-fold PBS were measured by accumulation of ten scans per sample in 1 mm path-length quartz cuvettes at 25 °C. To calculate the mean residue ellipticity θ_MRW_ in deg × cm^2^ × dmol^−1^ the following formula was used: θ_MRW_ = (θ_obs_ × MRW)/(c × d × 10), where θ_obs_ observed ellipticity (in degrees); c, concentration (in g/ml); d, cell path length (in cm), MRW (mean residue weight), molecular weight divided by number of peptide bonds.

### Modeling

IL-12Rβ2 domains D1 to D3 (UniProtKB - Q99665) and murine p40 (UniProtKB - P43432) were modeled using the Phyre2 web portal for protein modeling, prediction, and analysis (27). For modeling of the IL-12 signaling complex, a model of IL-12Rβ2 domains D1-D3 and the structure of hIL-12 (PDB 3HMX) were superpositioned using UCSF Chimera (28) onto the crystal structure of the IL-23:IL-23R:IL-12Rβ1 complex (6WDQ). To visualize the effect of p40D36K/W37K/Y38K mutation, these residues were mutated *in silico* using UCSF Chimera. Superpositioning of hp40 (PDB 5mzv) and a model of mp40 was done in UCSF Chimera.

## Data availability

All data are contained within the article.

## Supporting information

This article contains supporting information (18, 20).

## Conflict of interest

The authors declare that they have no conflicts of interest with the contents of this article. All authors have read the manuscript and agreed to the final version.
