# Supplementary material for: Tryptophan (W) at position 37 of murine IL-12/IL-23 p40 is mandatory for binding to IL-12Rβ1 and subsequent signal transduction
Source: J Biol Chem. 2021 Oct 9;297(5):101295. doi: 10.1016/j.jbc.2021.101295 (PMC8571081; doi:10.1016/j.jbc.2021.101295)
Supplement: Supplemental Figures S1–S6 [file mmc1.pdf]

Tryptophan (W) at position 37 of murine IL-12/IL-23 p40 is mandatory for binding to IL-12Rβ1 and subsequent signal transduction

Jacqueline Georgy[1], Yvonne Arlt[1], Jens M. Moll[1], Meryem Ouzin[1], Hendrik T. Weitz[1], Lothar Gremer[2, 3], Dieter Willbold[2, 3], Joachim Grötzinger[4], Felix Thives-Kurenbach[1], Jürgen Scheller[1] and Doreen M. Floss[1*]

From the [1]Institute of Biochemistry and Molecular Biology II, Medical Faculty, Heinrich-Heine-University Düsseldorf, Düsseldorf, Germany, [2]Institute of Biological Information Processing (IBI-7: Structural Biochemistry) and JuStruct: Jülich Center for Structural Biology, Forschungszentrum Jülich, Jülich, Germany, [3]Institut für Physikalische Biologie, Heinrich-Heine-Universität Düsseldorf, Düsseldorf, Germany and the [4]Institute of Biochemistry, Medical Faculty, Christian-Albrechts-University Kiel, Kiel, Germany

Supporting Information

Figure S1: Alignment of murine and human p40.

Figure S2: Dose-response analysis of HIL-23 variants on cellular proliferation of Ba/F3-gp130-mIL-12Rβ1-mIL-23R cells

Figure S3: Purification of cytokines using Strep-Tactin®XT 4Flow® columns.

Figure S4: Far-UV CD spectroscopy of HIL-23 and HIL-12 variants

Figure S5: Receptor binding of HIL-23 and HIL-12 variants.

Figure S6: Interaction of cytokines with respective receptors.

A

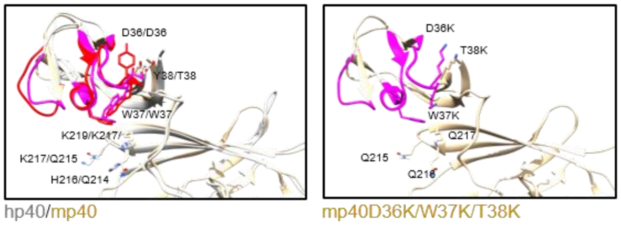

B

```
                                      ΔM23-D29  ΔY31-T38 W37
P43432|p40 MOUSE MCPQKLTISWFAIVLLVSPLMAMWELEKDVYVVEVDWTPDAPGETVNLTCDTPEEDDITW 60
P29460|p40 HUMAN MCHQQLVISWFSLVFLASPLVAIWELKKDVYVVELDWYPDAPGEMVVLTCDTPEEDGITW 60
                 ** *:*.****::*:*.***:*:***:*******:** ****** * *********.***
                                                P39 D40 loop 1 (W38-M46)
                                  E81 F82
P43432|p40 MOUSE TSDQRHGVIGSGKTLTITVKEFLDAGQYTCHKGGETLSHSHLLLHKKENGIWSTEILKNF 120
P29460|p40 HUMAN TLDQSSEVLGSGKTLTIQVKEFGDAGQYTCHKGGEVLSHSLLLLHKKEDGIWSTDILKDQ 120
                 * **   *:******** **** ************.**** *******:*****:***:
              loop 2 (L62-L69)  loop 3 (T76-Y88)

P43432|p40 MOUSE ---KNKTFLKCEAPNYSGRFTCSWLVQRNMDLKFNIKSSSSSPDSRAVTCGMASLSAEKV 177
P29460|p40 HUMAN KEPKNKTFLRCEAKNYSGRFTCWWLTTISTDLTFSVKSSRGSSDPQGVTCGAATLSAERV 180
                    ******:*** ******** **.  . **.*.:*** .* * :.**** *:****:*

P43432|p40 MOUSE TLDQRDYEKYSVSCQEDVTCPTAEETLPIELALEARQQNKYENYSTSFFIRDIIKPDPPK 237
P29460|p40 HUMAN RGDNKEYE-YSVECQEDSACPAAEESLPIEVMVDAVHKLKYENYTSSFFIRDIIKPDPPK 239
                   *:::** ***.**** :**:***:****: ::* :: *****::**************
                                                H216 K217 K219

P43432|p40 MOUSE NLQMKPLKNS-QVEVSWEYPDSWSTPHSYFSLKFFVRIQRKKEKMKETEEGCNQKGAFLV 296
P29460|p40 HUMAN NLQLKPLKNSRQVEVSWEYPDTWSTPHSYFSLTFCVQVQGKSKREKK--------DRVFT 291
                 ***:****** **********:**********.* *::* *.:: *:        . .:.

P43432|p40 MOUSE EKTSTEVQC-KGGNVCVQAQDRYYNSSCSKWACVPCRVRS 335
P29460|p40 HUMAN DKTSATVICRKNASISVRAQDRYYSSSWSEWASVPCS--- 328
                 :***: * * *...:.*:******.** *:**.***
```

Figure S1: Alignment of murine and human p40. A, Superpositioning of human p40 (hp40) (PDB 6WDQ) and a model of murine p40 (mp40). Ustekinumab binding epitope is highlighted (red). Corresponding residues of mIL-12p40 are indicated (magenta). For comparison, a model of mp40D36K/W37K/T38K is displayed in an additional panel. B, alignment of murine and human p40. Signal peptides M1 to A22 are underlined. N-terminal deletion variant ΔM23-D29 and ΔY31-T38 are highlighted in green and blue. Three loops (W38-M46, L62-L69 and T76-Y88) contribute to the binding epitope of Ustekinumab (1). Hypothesized residues in D1 of human p40, which might be important for binding to IL-12Rβ1 are shown in red Glassman 2021. Alanine substitutions, presented in purple, have been introduced into two loops of p40, which reduced the potency of IL-12 and IL-23 (2).

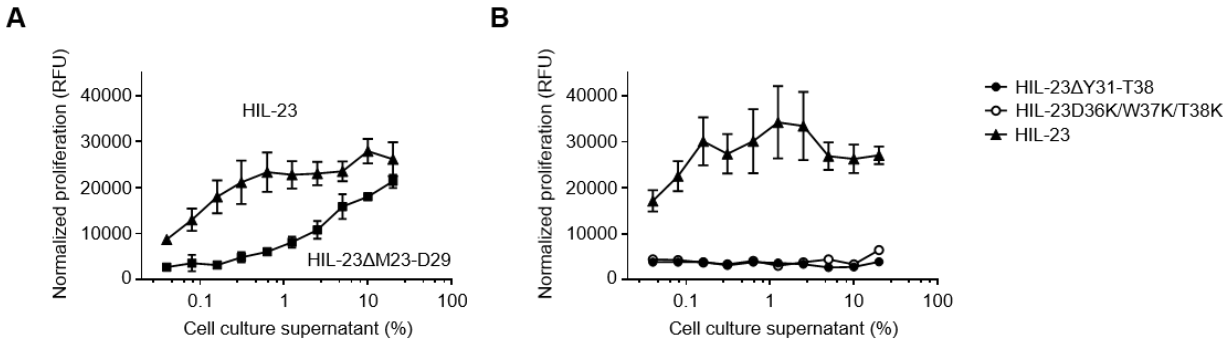

Figure S2: Dose-response analysis of HIL-23 variants on cellular proliferation of Ba/F3-gp130-mIL-12Rβ1-mIL-23R cells. The cells were cultured for 3 days in the presence of increasing concentrations of the indicated cytokines (0.04 to 20% conditioned cell culture supernatant of transfected CHO-K1 cells). The results of one representative experiment of three (A) or two (B) are shown. Error bars represent S.D. for technical replicates.

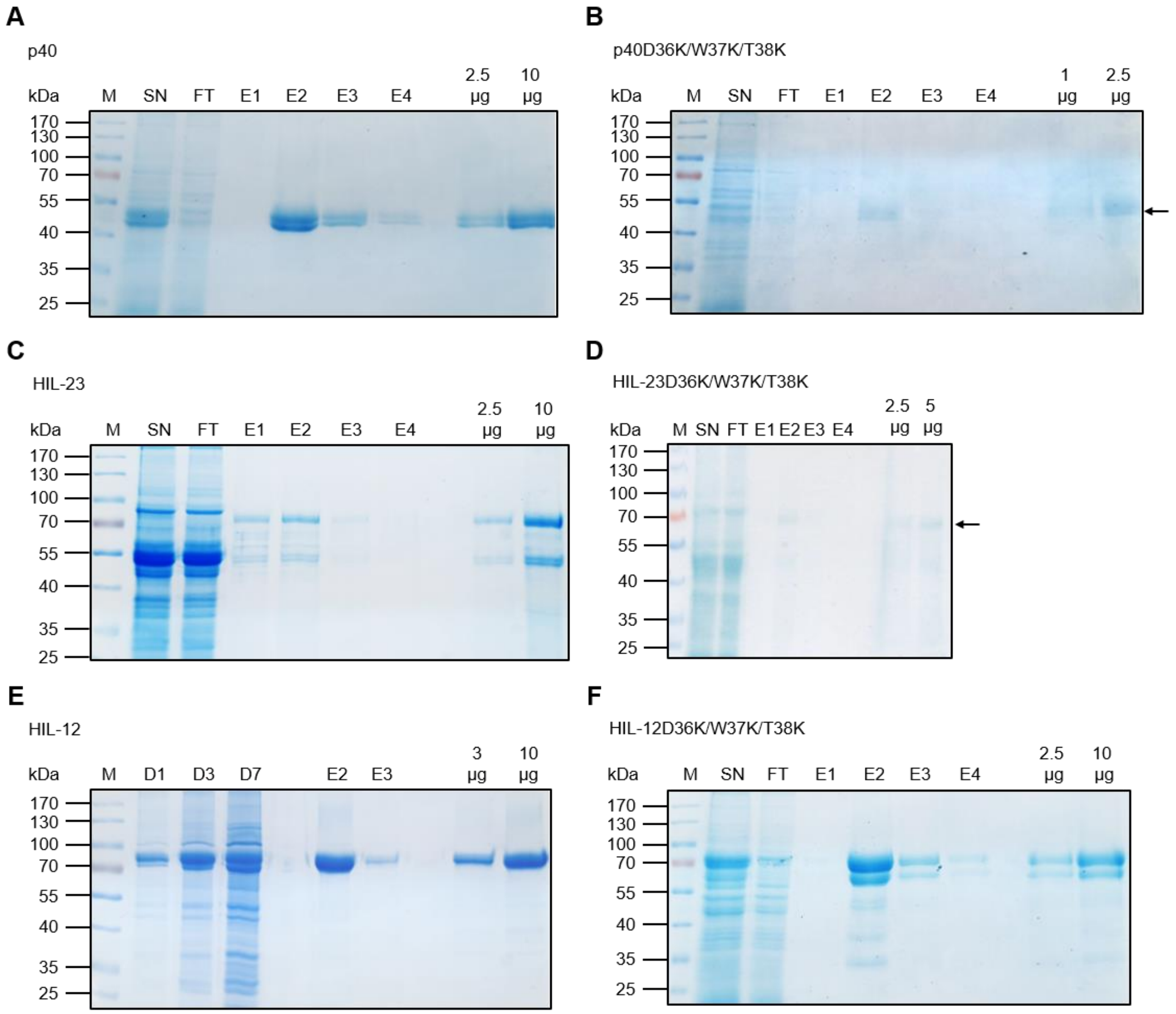

Figure S3: Purification of cytokines using Strep-Tactin®XT 4Flow® columns. Purity of the Expi cell produced murine p40, p40D36K/W37K/T38K, HIL-12, HIL-12D36K/W37K/T38K, HIL-23 and HIL-23D36K/W37K/T38K was analyzed by SDS-PAGE on reducing gels via Coomassie brilliant blue staining. M, molecular weight marker; SN, cell culture supernatant; FT, flow through; E1-E4, elution fraction 1 to 4; D1, D3, D7, cell culture supernatant of Expi cells from day 1, 3 or 7.

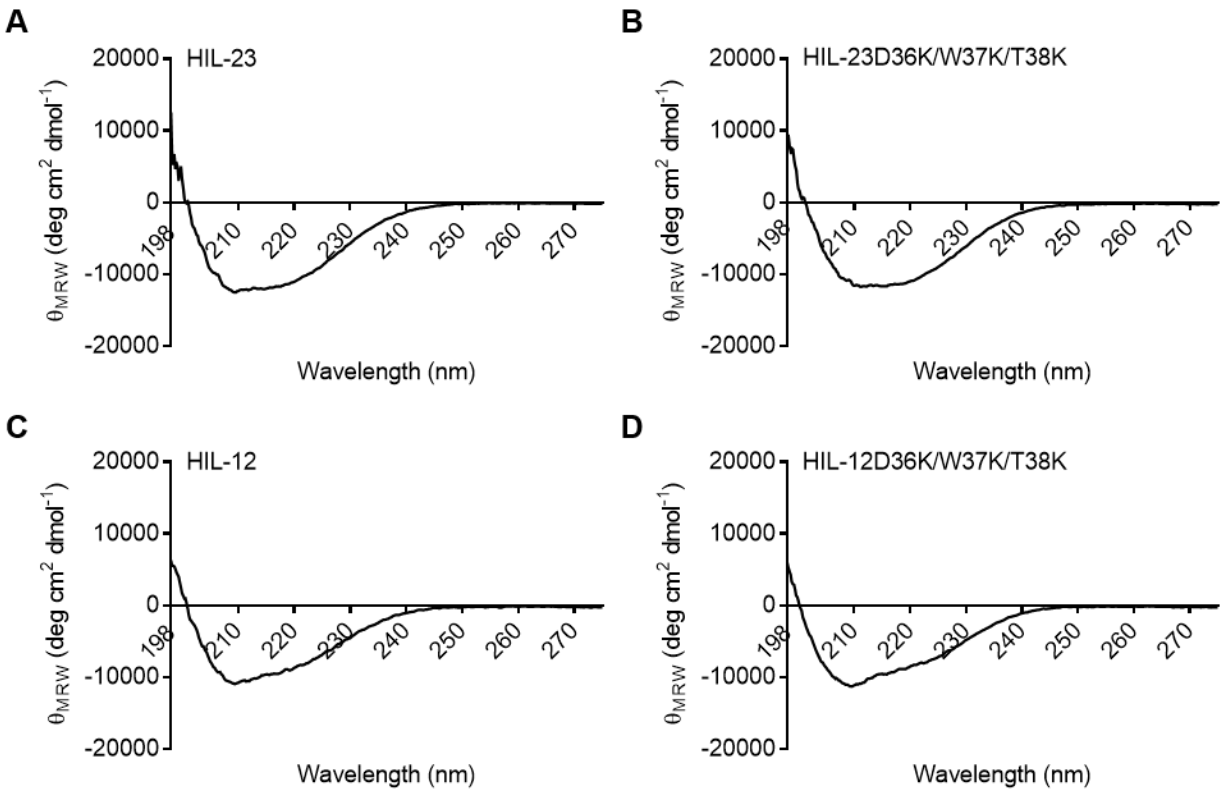

Figure S4: Far-UV CD spectroscopy of HIL-23 and HIL-12 variants. The far-UV spectra of HIL-23 (A) and HIL-23D36K/W37K/T38K (B), or HIL-12 (C) and HIL-12D36K/W37K/T38K (D) indicate that the wild type proteins and their KKK mutants have a highly similar overall secondary structure. Y-axis, θ_{MRW} in deg x cm^2 x $dmol^{-1}$; x-axis, wavelength in nm.

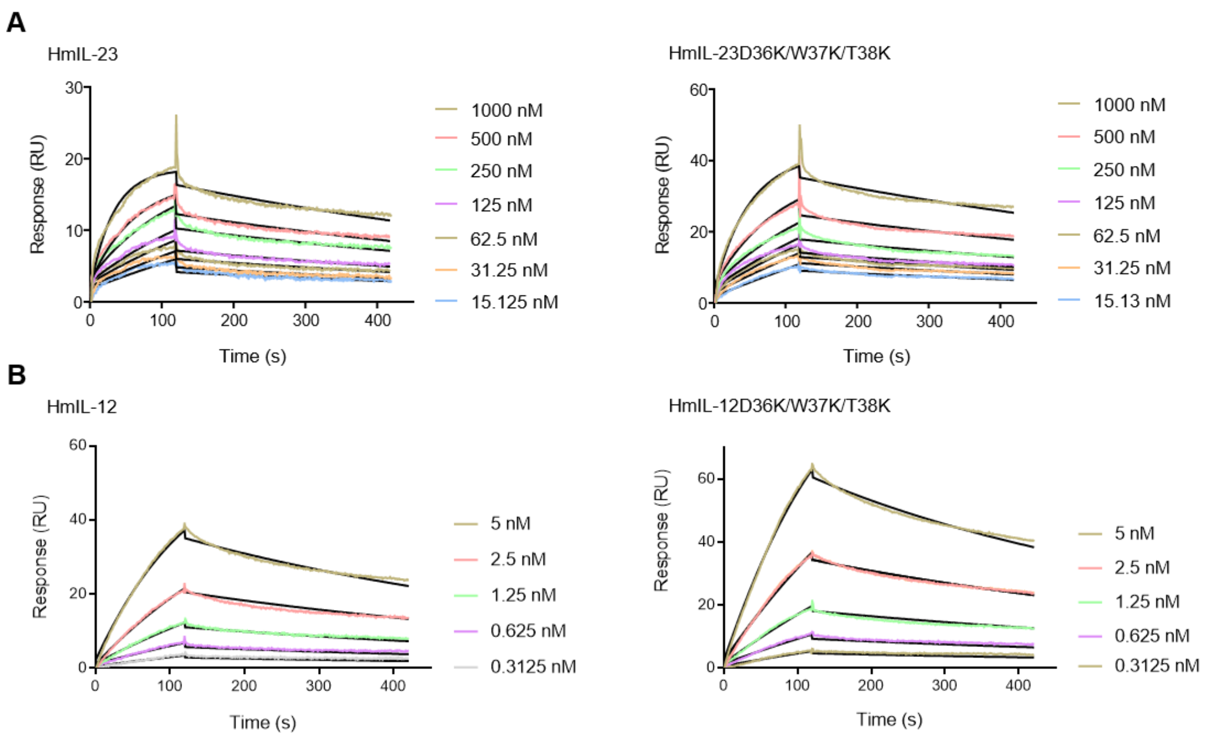

Figure S5. Receptor binding of HIL-23 and HIL-12 variants. A, surface plasmon resonance analysis of HIL-23 binding to IL-23R-Fc. IL-23R-Fc was captured on a ProtA chip and increasing concentrations of HIL-23 and HIL-23D36K/W37K/T38K were injected (15-1000 nM). B, surface plasmon resonance analysis of HIL-12 binding to IL-12Rβ2-Fc. IL-12Rβ2-Fc was captured on a ProtA chip and increasing concentrations of HIL-12 and HIL-12D36K/W37K/T38K were injected (0.3-5 nM). Sensorgrams in response units (RU) over time are depicted as colored lines, fit data are displayed as black lines.

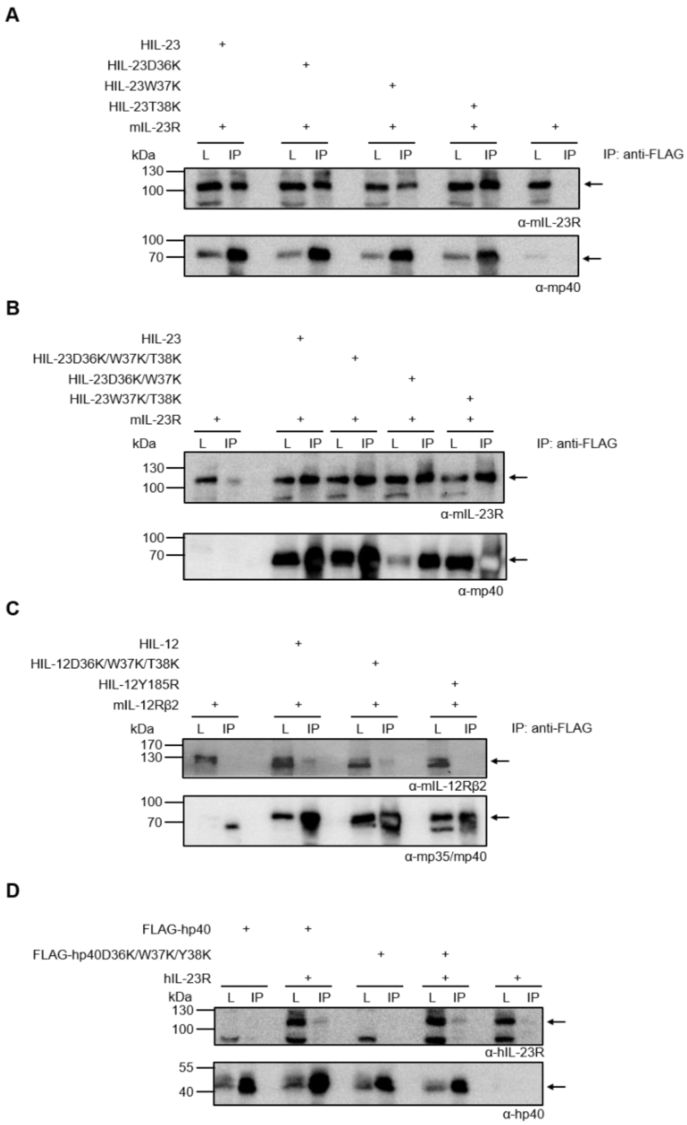

Figure S6: Interaction of cytokines with respective receptors. A, co-IP of FLAG-tagged murine HIL-23 variants (wild-type, D36K, W37K and T38K) and full-length mIL-23R. The position of mIL-23R and HIL-23 variants is indicated by arrows. One of two independent experiments is shown. B, co-IP of FLAG-tagged murine HIL-23 variants (wild-type, D36K/W37K/T38K, D36K/W37K, W37K/T38K) and full-length mIL-23. The position of mIL-23R and HIL-23 variants is indicated by arrows. One of two independent experiments is shown. C, co-IP of FLAG-tagged murine HIL-12 variants (wild-type, D36K/W37K/T38K, Y185R) and full-length mIL-12Rβ2. The position of mIL-12Rβ2 and HIL-12 variants is indicated by arrows. One of two independent experiments is shown. D, co-IP of FLAG-tagged human p40 variants (wild-type, D36K/W37K/Y38K) and full-length hIL-23R. The position of hIL-23R and hp40 variants is indicated by arrows. One of two independent experiments is shown.

References

1. Luo, J., Wu, S. J., Lacy, E. R., Orlovsky, Y., Baker, A., Teplyakov, A., Obmolova, G., Heavner, G. A., Richter, H. T., and Benson, J. (2010) Structural basis for the dual recognition of IL-12 and IL-23 by ustekinumab. *J. Mol. Biol.* **402**, 797-812

2. Glassman, C. R., Mathiharan, Y. K., Jude, K. M., Su, L., Panova, O., Lupardus, P. J., Spangler, J. B., Ely, L. K., Thomas, C., Skiniotis, G., and Garcia, K. C. (2021) Structural basis for IL-12 and IL-23 receptor sharing reveals a gateway for shaping actions on T versus NK cells. *Cell* **184**, 983-999 e924
